# Supplementary material for: Splice-Junction-Based Mapping of Alternative Isoforms in the Human Proteome
Source: Cell Rep. Author manuscript; Available in PMC 2020 Jan 15. (PMC6961840; doi:10.1016/j.celrep.2019.11.026)
Supplement: 2 [file NIHMS1546469-supplement-2.zip › DF1/PXD010154/Esophagus/Esophagus_6_MAP4_KKPCSETSQIEGSPTEF.pdf]

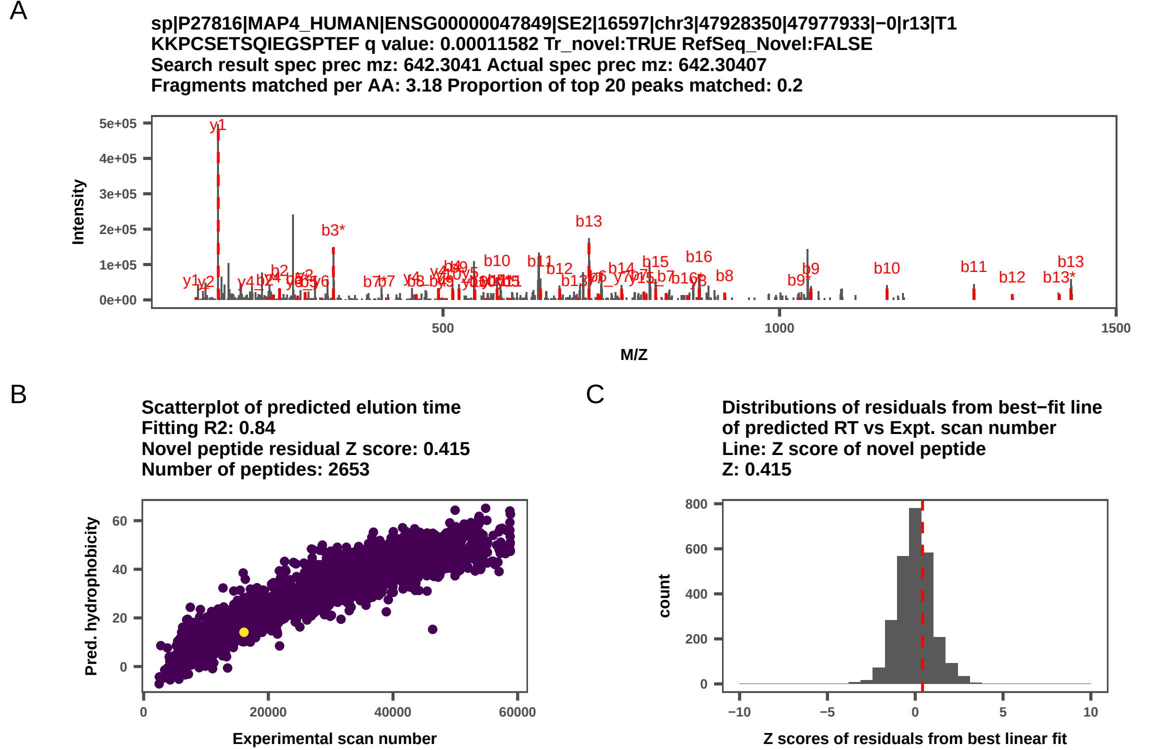
A
sp|P27816|MAP4_HUMAN|ENSG00000047849|SE2|16597|chr3|47928350|47977933|−0|r13|T1
KKPCSETSQIEGSPTEF q value: 0.00011582 Tr_novel:TRUE RefSeq_Novel:FALSE
Search result spec prec mz: 642.3041 Actual spec prec mz: 642.30407
Fragments matched per AA: 3.18 Proportion of top 20 peaks matched: 0.2
Intensity
5e+05
4e+05
3e+05
2e+05
1e+05
0e+00
y1
b3*
b13
b10
b11
b12
b15
b16
b9
b9*
b8
b10
b11
b12
b13
b13*
500
1000
1500
M/Z
B
Scatterplot of predicted elution time
Fitting R2: 0.84
Novel peptide residual Z score: 0.415
Number of peptides: 2653
Pred. hydrophobicity
60
40
20
0
0
20000
40000
60000
Experimental scan number
C
Distributions of residuals from best−fit line
of predicted RT vs Expt. scan number
Line: Z score of novel peptide
Z: 0.415
count
800
600
400
200
0
−10
−5
0
5
10
Z scores of residuals from best linear fit
